# Increased Risk of Invasive Aspergillosis in Immunocompromised Patients With Persistent SARS-CoV-2 Viral Shedding >8 Weeks, Retrospective Case-control Study

**DOI:** 10.1093/ofid/ofae012

**Published:** 2024-01-09

**Authors:** Cléa Melenotte, Nathalie Chavarot, Anne-Sophie L’Honneur, Sylvain Bodard, Morgane Cheminant, Adrien Flahault, Yann Nguyen, Marianne Burgard, Eric Dannaoui, Marie-Elisabeth Bougnoux, Perrine Parize, Claire Rouzaud, Anne Scemla, Etienne Canouï, Emmanuel Lafont, Damien Vimpere, Julien Zuber, Caroline Charlier, Felipe Suarez, Dany Anglicheau, Olivier Hermine, Fanny Lanternier, Luc Mouthon, Olivier Lortholary

**Affiliations:** Department of Infectious Diseases and Tropical Medicine, Hospital Necker-Enfants Malades, Public Assistance of the Hospital of Paris, Paris, France; Department of Nephrology and Kidney Transplantation, Hospital Necker-Enfants Malades, Public Assistance of the Hospital of Paris, Paris, France; Paris-Cité University, Paris, France; Department of Nephrology and Kidney Transplantation, European Hospital Georges Pompidou, Public Assistance of the Hospital of Paris, Paris, France; Paris-Cité University, Paris, France; Department of Virology, Cochin University Hospital, Public Assistance of the Hospital of Paris, Paris, France; Paris-Cité University, Paris, France; Department of Imaging, Hospital Necker-Enfants Malades, Public Assistance of the Hospital of Paris, Paris, France; Paris-Cité University, Paris, France; Department of Hematology, Hospital Necker-Enfants Malades, Public Assistance of the Hospital of Paris, Paris, France; Department of Nephrology and Kidney Transplantation, European Hospital Georges Pompidou, Public Assistance of the Hospital of Paris, Paris, France; Department of Internal Medicine, University Hospital Cochin, Public Assistance of the Hospital of Paris, Paris, France; Department of Virology, Hospital Necker-Enfants Malades, Public Assistance of the Hospital of Paris, Paris, France; Paris-Cité University, Paris, France; Department of Mycology and Parasitology, Hospital Necker-Enfants Malades, Public Assistance of the Hospital of Paris, Paris, France; Paris-Cité University, Paris, France; Department of Mycology and Parasitology, Hospital Necker-Enfants Malades, Public Assistance of the Hospital of Paris, Paris, France; Department of Infectious Diseases and Tropical Medicine, Hospital Necker-Enfants Malades, Public Assistance of the Hospital of Paris, Paris, France; Department of Infectious Diseases and Tropical Medicine, Hospital Necker-Enfants Malades, Public Assistance of the Hospital of Paris, Paris, France; Department of Nephrology and Kidney Transplantation, Hospital Necker-Enfants Malades, Public Assistance of the Hospital of Paris, Paris, France; Mobile Team of Infectious Diseases and Tropical Medicine, Cochin University Hospital, Public Assistance of the Hospital of Paris, France; Department of Internal Medicine, European Hospital Georges Pompidou, Public Assistance of the Hospital of Paris, Paris, France; Department of Intensive Care Unit, Hospital Necker-Enfants Malades, Public Assistance of the Hospital of Paris, Paris, France; Department of Nephrology and Kidney Transplantation, Hospital Necker-Enfants Malades, Public Assistance of the Hospital of Paris, Paris, France; Paris-Cité University, Paris, France; Paris-Cité University, Paris, France; Mobile Team of Infectious Diseases and Tropical Medicine, Cochin University Hospital, Public Assistance of the Hospital of Paris, France; Paris-Cité University, Paris, France; Department of Hematology, Hospital Necker-Enfants Malades, Public Assistance of the Hospital of Paris, Paris, France; Department of Nephrology and Kidney Transplantation, Hospital Necker-Enfants Malades, Public Assistance of the Hospital of Paris, Paris, France; Paris-Cité University, Paris, France; Paris-Cité University, Paris, France; Department of Hematology, Hospital Necker-Enfants Malades, Public Assistance of the Hospital of Paris, Paris, France; Department of Infectious Diseases and Tropical Medicine, Hospital Necker-Enfants Malades, Public Assistance of the Hospital of Paris, Paris, France; Paris-Cité University, Paris, France; Paris-Cité University, Paris, France; Department of Internal Medicine, University Hospital Cochin, Public Assistance of the Hospital of Paris, Paris, France; Department of Infectious Diseases and Tropical Medicine, Hospital Necker-Enfants Malades, Public Assistance of the Hospital of Paris, Paris, France; Paris-Cité University, Paris, France; Mycology Department, Institut Pasteur, Université Paris Cité, National Reference Center for Invasives Mycoses and Antifungals, Mycology Translational Research Group, Paris, France

**Keywords:** COVID-19, death, invasive aspergillosis, persistent viral shedding, immunocompromised host, solid organ transplant recipients, hematological diseases, SARS-CoV-2

## Abstract

**Background:**

Immunocompromised patients now represent the population most at risk for severe coronavirus disease 2019. Persistent severe acute respiratory syndrome coronavirus 2 (SARS-CoV-2) viral shedding was reported in these patients ranging from several weeks up to 9 months. We conducted a bicentric retrospective case-control study to identify risk and prognostic factors associated with persistent viral shedding in immunocompromised patients.

**Material and Methods:**

Symptomatic immunocompromised adults with persistent SARS-CoV-2 viral shedding >8 weeks were retrospectively included between 1 March 2020 and 24 April 2022 at 2 university hospitals in Paris, France, and matched with a control group consisting of symptomatic immunocompromised patients without persistent viral shedding.

**Results:**

Twenty-nine immunocompromised patients with persistent viral shedding were compared with 40 controls. In multivariate analysis, fever and lymphocytopenia (<0.5 G/L) were associated with an increased risk of persistent viral shedding (odds ratio [OR]: 3.3; 95% confidence interval [CI], 1.01–11.09) *P* = .048 and OR: 4.3; 95% CI, 1.2–14.7; *P* = .019, respectively). Unvaccinated patients had a 6-fold increased risk of persistent viral shedding (OR, 6.6; 95% CI, 1.7–25.1; *P* = .006). Patients with persistent viral shedding were at risk of hospitalization (OR: 4.8; 95 CI, 1.5–15.6; *P* = .008), invasive aspergillosis (OR: 10.17; 95 CI, 1.15–89.8; *P* = .037) and death (log-rank test <0.01).

**Conclusions:**

Vaccine coverage was protective against SARS-CoV-2 persistent viral shedding in immunocompromised patients. This new group of immunocompromised patients with SARS-CoV-2 persistent viral shedding is at risk of developing invasive aspergillosis and death and should therefore be systematically screened for this fungal infection for as long as the viral shedding persists.

## Teaser

Immunocompromised patients are the population most at risk of severe coronavirus disease 2019. Among them, patients with persistent severe acute respiratory syndrome coronavirus 2 viral shedding (>8 weeks) have a 10-fold higher risk of invasive aspergillosis, 4-fold increased risk of hospitalization, and increased risk of death.

Three years after the start of the coronavirus disease 2019 (COVID-19) pandemic, in December 2023, the World Health Organization reported 772 million and 6.9 million cumulative cases and death, respectively [1, 2]. Immunocompromised (IC) patients now compose the population the most at risk for severe COVID-19 (intensive care unit [ICU], hospitalization, and death) [3–6]. It represents at least 2%–3% of the US and EU population and includes patients with solid cancers and hematological malignancies, hematopoietic stem cell transplantation, solid organ transplant (SOT), inborn error of immunity, human immunodeficiency virus (HIV) (CD4^+^ T-cell count <200/mm^3^), autoimmune diseases, and patients under immunosuppressives therapies [7–9]. Because IC patients lack effective vaccine immune responses, whether humoral (in patients receiving B-cell–targeting therapies) or both humoral and cellular in SOT, a COVID-19 booster is recommended every 6 months to increase vaccinal response [7–10]. Nonetheless, it remains a heterogeneous group of patients, in which the immunological vaccine response is largely uncertain, varying from 1 patient to another [8]. Monoclonal antibody prophylaxis has therefore been an effective alternative for patients who do not develop postvaccinal immune responses, with encouraging results but that are limited by the emergence of new resistant variants of severe acute respiratory syndrome coronavirus 2 (SARS-CoV-2) [11, 12].

Since the beginning of the pandemic, persistent viral shedding has been reported in IC patients varying from several weeks up to 9 months and new challenges have been raised [13–16]. First, persistent contagiousness prompted the authorities to extend eviction until 20 days in these patients [17]. Second, they were subject to prolonged hospitalization, late deterioration, and complications sometimes favored by the intensification of the immunosuppressive drugs to treat the underlying disease(s). Finally, persistent viral shedding in this group of patients raised major therapeutic concerns because antiviral drugs could not eradicate the virus and were responsible for the emergence of mutations, resistance, and new variants [13, 18–20]. Several risk factors for persistent viral shedding had been identified, among which are B-cell dysfunction (malignant lymphoma, multiple myeloma, anti-CD20 and anti-CD19 therapies), lymphocytopenia, serum elevation of type 1 interferon, immature neutrophils, delayed antiviral therapy, and D-dimer elevation [15, 16, 21–23]. Persistent viral shedding was associated with a spike protein mutation and an increased risk of bacterial infections and death [15, 16, 21–23]. However, the absence of a standardized definition of the duration of “persistence” makes it difficult to interpret and extrapolate previous reports [16].

We report here a bicentric retrospective case-control study to identify risk factors for persistent viral shedding and prognostic factors among IC adults.

## METHODS

### Study Design

We conducted a retrospective bicentric case control study including IC patients with persistent SARS-CoV-2 viral shedding >8 weeks. Patients were included from 2 different university hospitals, Cochin Hospital and Necker Enfants-Malades Hospital (“Paris-Centre hospital group”), and IC patients were included from the departments of infectious diseases and tropical medicine, nephrology, kidney transplantation, hematology, ICU, and internal medicine. The study was approved by the French Assistance Publique des Hôpitaux de Paris Research Ethics Committee “Comité d’éthique de la recherche APHP centre” (institutional review board registration: #00011928), in accordance with current standards.

The management of patients with COVID-19 within the “Paris-Centre” hospital group was comparable and homogeneous in all the departments considered for this study. It followed the recommendations of the multidisciplinary consultation meetings held monthly within the hospital group during the pandemic period and chaired by one of us (L. M.).

### Definition of Patients

Persistent viral shedding was defined by the presence of repeated (≥2) positive SARS-CoV-2 polymerase chain reaction (PCR) tests on a nasopharyngeal sample for at least a period > 8 weeks without a negative nasopharyngeal SARS-CoV-2 PCR during this period. All patients with persistent viral shedding from 1 March 2020 to 24 April 2022 were included. Data collection included clinical and biological presentations at diagnosis, SARS-CoV-2 genotype, current treatment, specific anti–COVID-19 medication, and potential complications, including documented bacterial and fungal infections and outcomes. Clinical complications such as COVID-19–related invasive aspergillosis and mucormycosis were identified among the included patients according to the current guidelines [24, 25]. Probable and proven invasive aspergillosis, regardless of the site of infection (pulmonary and/or other), in patients who were not admitted in ICU were considered according to the European Organization for Research and Treatment of Cancer guidelines [26, 27]. Messenger RNA vaccines (Pfizer and Moderna available in France) and monoclonal antibodies prophylaxis (sotrovimab, casirivimab/imdevimab, tixagevimab/cilgavimab) were considered when administered before COVID-19 infection. A centralized second computed tomography (CT) scan imaging reading was performed in all patients by a single qualified radiologist (S. B.). We included IC patients with primary immune deficiency/inborn errors immunity, HIV infection with CD4-T cells < 200/mm^3^, autoimmune diseases, SOT, allogeneic hematopoietic stem cell transplantation, chronic lymphoid malignancies (treated or untreated) (ie, chronic lymphocytic leukemia, non-Hodgkin lymphoma and myeloma), and patients receiving immunosuppressive therapies such as chimeric antigen receptor–T-cell therapy or therapeutic bispecific antibodies, anti-CD20 antibodies or Bruton tyrosine kinase inhibitors, azathioprine, cyclophosphamide, methotrexate, mycophenolate mofetil, calcineurin inhibitors, corticosteroids, and belatacept.

Controls (n = 40) were selected as follows: (1) between 1 March 2020 and 24 April 2022 (during the same period as the cases); (2) were immunocompromised patients corresponding to the criteria defined previously; (3) were immunocompromised patients with symptomatic COVID-19 infection; (4) had proven positive SARS-CoV-2 PCR on nasopharyngeal specimen; (5) and proven negative for SARS-CoV-2 on PCR via nasopharyngeal specimen within ≤8 weeks after the first positive test; and (6) were followed up > 6 months in our centers with regular visits because of their immunocompromised status.

We excluded asymptomatic patients, patients for whom clinical data were unavailable, patients with reinfection (defined as negative PCR between 2 positive results or identification of 2 different SARS-CoV-2 variants), and patients who did not had a 6-month follow-up in our centers.

### Statistical Analysis

To compare the distribution of continuous and dichotomous variables between 2 groups, we used the χ^2^ or the 2-sided Fisher exact tests, respectively. All tests were 2-sided, and *P* < .05 was considered significant. Indicative factors for persistent viral shedding, death, and invasive aspergillosis (IA) were determined using the logistic regression model. The Kaplan-Meier method was used to determine cumulative probabilities of all-cause death and cumulative probabilities of IA, with between-group comparisons of cumulative event rates calculated using the log-rank test. All analyses were performed using SPSS version 29.0 statistical software.

## RESULTS

**Figure 1.**
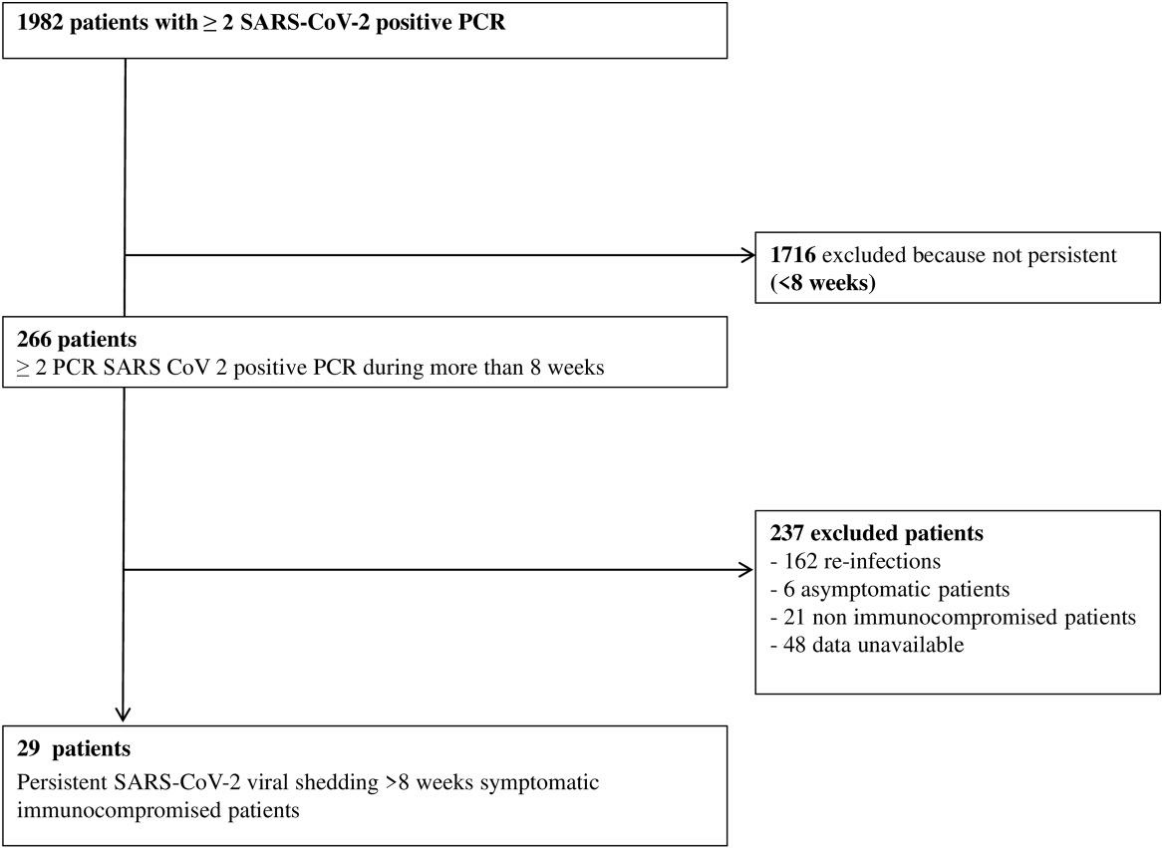
Flow chart.

During the study period, 1982 patients had a positive SARS-CoV-2 PCR result on nasopharyngeal samples at Cochin Hospital-University and 521 at Necker Enfants-Malades Hospital-University. Of these, 266 had at least 2 positive SARS-CoV-2 PCRs on nasopharyngeal samples over 8 weeks. A total of 237 were excluded because of reinfection (ie, negative PCR between 2 samples or identification of 2 different variants), because they were asymptomatic (n = 6), non-IC patients (n = 21), or because of unavailable data (n = 48). Overall, the incidence of persistent viral shedding > 8 weeks from those 2 centers was 1.4% (Figure 1).

### Characteristics of Patients With Persistent Viral Shedding (>8 Weeks)

We identified 29 patients with persistent viral shedding. The mean age was 57.7 ± 16 years and 55.2% were men. Most patients, 55.2% (n = 16) were SOT recipients, including 13 kidney transplant recipients (KTRs), 1 kidney and liver transplant recipient, and 2 lung transplant recipients. Ten patients presented hematological diseases (Table 1; Supplementary Figure 1). Two patients had concomitant SOT and hematological disease, including 1 KTR with a diffuse and large B-cell lymphoma and 1 lung transplant recipient with acute myeloid leukemia. One patient with HIV developed lymphoma and 4 had other immunodeficiencies (Table 1; Supplementary Figure 1). Ninety-three percent of these patients (n = 27) were receiving immunosuppressive drugs at the time of diagnosis and 46% (n = 22) had received long-term corticosteroid therapy (Table 1). Five (17%) presented with obesity and 4 (13%) with undernutrition (body mass index <18.5 kg/m^2^). Twelve patients (41%) had hypertension and 8 (27%) had diabetes. Fifty-one percent of patients (n = 14/27) were vaccinated before having persistent SARS-CoV-2 infections and only 27.6% (n = 8/27) received at least 3 doses of vaccine (data were not available for 2 patients). Twenty percent of these patients (6/29) received monoclonal antibody prophylaxis before infection. At the time of COVID-19 diagnosis, clinical presentation was dominated by fever (n = 20, 69%), pulmonary manifestations (n = 20, 69% [cough, 62%; dyspnea, 48%]), and asthenia (n = 18, 62%). Most of the patients (n = 22, 76%) had lymphopenia (<1 G/L), 55% (n = 16) had severe lymphocytopenia (<0.5 G/L), 52% (n = 14/27) had hypogammaglobulinemia at the time of diagnosis, and 75% (n = 21) presented with an inflammatory syndrome (C-reactive protein >10 mg/L at the time of SARS-CoV-2 diagnosis). Characteristics of patients and underlying conditions are described in Table 1 and Supplementary Figure 1. The mean duration in viral shedding was 129 ± 80.74 days (median: 108; interquartile range, 77–176.5 days). No significant difference of viral shedding duration was observed between the subgroups of patients (ie, between SOT and those with hematological disorders) (Supplementary Figure 1). The mean duration of symptoms was 137 ± 83 days in IC patients and 16 ± 16 days in the control group. Omicron variant was the most represented (n = 13, 59%), followed by Alpha (n = 6, 27.2%) and Delta (n = 3, 13.6%); data were not available for 24% of patients (n = 7). Fifty-eight percent of patients were hospitalized (n = 17), including 5 in the ICU (17%), 11 (37%) required oxygen therapy, and 23 (79%) received anti–COVID-19 specific treatment including dexamethasone in 10 (34%), monoclonal antibodies in 12 (41%), and plasma therapy in 11 (38%) (Table 1). Three patients received dexamethasone in addition to monoclonal antibodies and plasma therapy. The outcome was marked by the occurrence of documented bacterial infection in 7 patients (24%), IA in 6 (20%), and death in 8 (27%) (Tables 1–3).

**Table 1.** Characteristics of Patients in Persistent Viral Shedding and Control Groups (Univariate Analysis)

Characteristics of Patients	Persistent Viral Shedding (n = 29)		Controls (n = 40)		*P*
Sex (M)	16 (55.2%)		21(52.5%)		.8
Age (mean ± SD)	57.72 ± 16.36		57.15 ± 16		.5
Medical history					
SOT	16/29	55.2%	30/40	75%	.08
Malignant hemopathy	10/29	34.5%	9/40	22.5%	.2
ANCA-vasculitis	1/29	3.4%	0/40	0%	.5
Inborn error immunity	1/29	3.4%	3/40	7.5%	.6
COPD and cortico-dependent asthma	1/29	3.4%	0/40	0%	.5
Spondyloarthritis and Crohn disease	1/29	3.4%	0/40	0%	.5
HIV	2/29	6.9%	0/40	0%	.1
Obesity (BMI > 30 kg/m^2^)	5/29	17.2%	6/40	15%	1.0
Undernutrition (BMI < 18.5 kg/m^2^)	4/29	13.8%	2/40	5%	.2
Hypertension	12/29	41%	23/40	57%	.1
Diabetes	8/29	27%	10/40	25%	.8
Immunosuppressive drugs	**27/29**	**93.1%**	**33/40**	**82.5%**	.1
Glucocorticoids	22/29	46.8%	25/40	52.3%	.2
Chemotherapy	3/29	10.3%	1/40	2.5%	.3
Tacrolimus	11/29	37.9%	22/40	55%	.1
MMF	13/29	44.8%	21/40	52.5%	.6
Everolimus	2/29	6.9%	1/40	2.5%	.5
Methotrexate	1/29	3.4%	0/40	0%	.4
Cyclosporine	1/29	3.4%	4/40	10%	.3
Belatacept	3/29	10.3%	2/40	5%	.6
Venetoclax	3/29	10.3%	1/40	2.5%	.3
Azathioprine	0/29	0%	3/40	7.5%	.2
Tyrosine kinase inhibitor	3/29	10.3%	1/40	2.5%	.3
Anti-CD20	5/29	17.2%	1/40	2.5%	.07
Prophylaxis against SARS-CoV-2 before COVID-19 infection					
Vaccination before COVID-19	**14/27**	**51.9%**	**33/39**	**84.6%**	**.004**
≥3 doses	**8 /27**	**27.6%**	**25/36**	**69.44%**	**.007**
Monoclonal Ab prophylaxis	6/29	20.7%	3/37	8.1%	.1
Biology at the time of diagnosis					
First PCR (Ct value)	21.26 ± 6.2	…	22.4 ± 6.9	…	.7
Lymphopenia < 1 G/L	22/29	75.9%	21/37	56.7%	.1
Lymphopenia < 0.5 G/L	**16/29**	**55.2%**	**10/37**	**27%**	**.02**
Hypogammaglobulinemia	14/27	51.8%	20/28	71%	.2
CRP > 10 mg/L	21/28	75%	19/36	52.7%	.1
Duration of SARS-CoV-2 viral shedding (d)	129 ± 80,74		23 ± 16		**<.01**
Variant					
Alpha	6/22	27.2%	0/31	0%	**.008**
Beta	0/22	0%	2/31	6.4%	.6
Delta	3/22	13.6%	9/31	29%	.3
Omicron	13/22	59%	21/31	65.6%	.8
Clinical symptoms					
Duration of clinical symptoms	137 ± 83 d		16.5 ± 16.8 d		**<.001**
ENT	**11/29**	**37.9%**	**26/40**	**65%**	**.02**
Odynophagia	**1/29**	**3.4%**	**12/40**	**30%**	**.005**
Anosmia	3/29	10%	4/40	10%	1.0
Ageusia	3/29	10.3%	5/40	12.5%	1.0
Digestive	7/29	24.1%	9/40	22.5%	1.0
Pulmonary	20/29	69%	27/40	67.5%	.8
Cough	18/29	62.1%	26/40	65%	.8
Dyspnea	14/29	48.3%	13/40	32.5%	.1
Asthenia	18/29	62.1%	21/40	52.5%	.4
Myalgia	6/29	20.7%	10/40	25%	.7
**Fever**	**20/29**	**69%**	**17/40**	**42.5%**	**.03**
Weight loss	5/29	17.2%	2/40	5.1%	.1
Treatment					
Hospitalization	**17/29**	**58,6%**	**10/40**	**25%**	**.005**
ICU	5/29	17.2%	2/40	5%	.1
Death	**8/29**	**27%**	**0/40**	**0%**	**<.001**
IA	**6/29**	**20.6%**	**1/40**	**2.5%**	**.03**
IA 8 wks after COVID-19 diagnosis	**3/29**	**10.3%**	**0/40**	**0%**	**.03**
Bacterial documented infection	7/29	24.1%	4/40	10%	.1
Oxygen therapy	11/29	37.9%	7/40	17.5%	.09
Noninvasive mechanical ventilation	4/29	13.8%	1/40	2.5%	.5
Invasive mechanical ventilation	2/29	6.9%	0/40	0%	.1
Specific treatment	23/29	79.3%	31/40	77.5%	.8
Monoclonal antibodies	**12/29**	**41.4%**	**27/40**	**67.5%**	**.03**
Sotrovimab	7/29	24.1%	8/40	20%	.7
Casirivimab/imdevimab	**3/29**	**10.3%**	**14/40**	**35%**	**.02**
Tixagevimab/cilgavimab	3/29	10.3	5/40	12.5%	1.0
Tocilizumab	5/29	37.9%	2/40	11.5%	.1
Plasmatherapy	**11/29**	**39%**	**1/40**	**2.5%**	**<.001**
Nirmatrelvir/ritonavir	2/29	6.9%	0/40	0%	.1
Remdesivir	1/29	4.5%	1/40	2.5%	1.0
DXM	**10/29**	**34.4%**	**5/40**	**12.5%**	**.04**
DXM + monoclonal Ab	3/29	10.3%	2/40	5%	.7
DXM + plasmatherapy	**6/29**	**20.7%**	**0/40**	**0%**	**.003**
Monoclonal Ab + plasmatherapy	**5/29**	**17.2%**	**0**/40	**0%**	**.006**
DXM + nirmatrelvir/ritonavir	1/29	3.4%	0/40	0%	.4
DXM + remdesivir	1/29	3.4%	0/40	0%	.4
DXM + monoclonal Ab + plasmatherapy	**3/29**	**10.3%**	**0/40**	**0%**	**.03**

χ^2^ and Fisher exact test. Values in bold indicate significant results.

Abbreviations: Ab, antibody; ANCA, antineutrophil cytoplasm antibodies; BMI, body mass index; COPD, chronic obstructive pulmonary disease; CRP, C-reactive protein; DXM, dexamethasone; ENT, ear nose and throat; HIV, human immunodeficiency virus; IA, invasive aspergillosis; ICU, intensive care unit; M, male; MMF, mycophenolate mofetil; PCR, polymerase chain reaction; SD, standard deviation; SOT, solid organ transplantation.

**Table 2.** Invasive Aspergillosis in Immunocompromised Patients With Persistent Viral Shedding

Age/sex	Medical History	Prophylaxis Against COVID-19	Immunosuppressive Drugs at COVID-19 Infection	SARS-CoV-2 Viral Shedding (d)	Delay Between SARS-CoV-2 first + test—IA (d)	Possible/probable IA	Mycological Documentation	Treatment	Outcome
71/F	Kidney transplant	3 doses of vaccine Monoclonal Ab prophylaxis	Tacrolimus	60	36	Probable PIA (NA) (ICU->CAPA)	GM Ag +	Voriconazole -> Isavuconazole	Alive
81/F	Marginal zone lymphoma	2 doses of vaccine	Chlorambucil	105	9	Probable PIA	Blood PCR + sputum culture (*A. fumigatus*)	Isavuconazole-> Voriconazole	Alive
61/M	Lung transplant and acute leukemia	Nothing	Blinatumomab-ponatinib-tacrolimus-CTC	143	29	Proven chondro-sternal IA Probable PIA	Histology and culture (*A. flavus*) Sputum: *A. fumigatus*	AMBL Posaconazole	Alive
70/F	Mantle lymphoma	2 doses of vaccine	Ibrutinib and venetoclax	63	64	Probable PIA	BAL culture + PCR (*A. fumigatus*)	Voriconazole	ICU Died (85 d after IA)
40/M	Combined immunodeficiency with heterozygous POLA1 mutation	3 doses of vaccine	None	69	97	Probable PIA	BAL culture + PCR+ GM Ag (*A. fumigatus*)	Voriconazole-> AMBL	Died (125 d post IA)
55/F	Lung transplant	3 doses of vaccine Monoclonal Ab prophylaxis	Tacrolimus CTC MMF	416	366	Probable mucormycosis and PIA (ICU->CAPA)	BAL+ sputum culture + GM Ag+ Blood PCR (*A. fumigatus*) Blood Mucor+ culture biopsy of the left nostril (*Rhizopus arrhizus*)	Voriconazole -> AMBL	Alive

Abbreviations: Ab, antibody; AMBL, amphotericin B liposomal; BAL, bronchoalveolar lavage; CAPA, COVID-19–associated pulmonary aspergillosis; CLL, chronic lymphoblastic leukemia; CTC, corticosteroids; DLBCL, diffuse and large B-cell lymphoma; F, female; GM Ag, galactomannan antigen; M, male; MMF, mycophenolate mofetil; NA, not available; PCR, polymerase chain reaction; PIA, pulmonary invasive aspergillosis.

**Table 3.** Characteristics of Patients With Persistent Viral Shedding who Died (8/29)

Sex/age	Medical History	Immunosuppressive Drug	Prophylaxis Against COVID-19	COVID-19 Treatment	Complications Leading To Death	ICU Stay (d)	Duration And Delay (d)	
							SARS-CoV-2 VS	Delay between first SARS-CoV-2 PCR and death
M/65	Kidney transplant	Tacrolimus CTC MMF	Nothing	Oxygen/DXM/tocilizumab	ARDS (*P. aeruginosa* VAP) *C. difficile* infection; peritonitis	147	104	314
M/70	CLL	Ibrutinib	Nothing	Oxygen/DXM/plasmatherapy/tocilizumab	Richter syndrome/pancytopenia/hemorrhagic shock	39	212	220
F/79	DLBCL	Venetoclax-idelalisib	3 doses of vaccine	Plasmatherapy/monoclonal Ab	Colitis, pneumonia	No	184	490
M/50	Kidney transplant	CTC	Nothing	Oxygen/DXM/tocilizumab/plasmatherapy	ARDS (*P. aeruginosa* VAP)	81	114	115
F/70	Mantle lymphoma	Ibrutinib	2 doses of vaccine	Monoclonal Ab	ARDS + *P. aeruginosa* VAP Probable PIA	20	63	155
M/40	Combined immunodeficiency with heterozygous POLA1 mutation	None	3 doses of vaccine/monoclonal Ab	Monoclonal Ab (×2)/ plasmatherapy	*P. aeruginosa* septic shock + probable PIA	24	69	121
M/65	DLBCL	Venetoclax	Nothing	Oxygen/tocilizumab/plasmatherapy	DLBCL progression/possible PIA	No	279	748
M/89	Myelodysplastic syndrome	None	2 doses of vaccine	Oxygen/DXM	Septic shock *K. pneumonia*	No	65	479

Abbreviations: Ab, antibody; ARDS, acute respiratory distress syndrome; CLL, Chronic lymphoid leukemia; CTC, corticosteroids; DLBCL, diffuse and large B-cell lymphoma; DXM, dexamethasone; F, female; ICU, intensive care unit; M, male; MMF, mycophenolate mofetil; PIA, pulmonary invasive aspergillosis; VAP, ventilation-acquired pneumonia; VS, viral shedding.

### Radiological Evolution

A chest CT scan was performed in 17 patients at the time of diagnosis. Ten had minimal infiltrates (<10%), 1 had moderate (10%–25%), 2 had extensive (20%–50%), 2 had severe (50%–75%) infiltrates, and 2 had no initial lesions. Chest CT scans were performed in 16 of these 17 patients 2 months after the first positive SARS-CoV-2 PCR (1 patient did not have a CT scan). Eleven (68.75%) had persistent lung infiltrates. Five had minimal lung infiltrates, 1 moderate, 2 had extensive (but less on the previous CT), 2 had severe, and 1 worsened with critical lesions (>75%). The latter had primary minimal lesions and presented severe complications with IA and mucormycosis (Supplementary Figure 3).

### Complications, Invasive Aspergillosis and Death

Among the 29 patients with persistent viral shedding, 6 (20%) presented IA compared with 1 in the control group. The mean duration of SARS-CoV-2 viral shedding in these patients was 142 ± 137 days. The delay between first positive SARS-CoV-2 PCR and IA was 100 ± 133 days. All of them had invasive pulmonary aspergillosis. One patient developed proven chondrosternal IA (he received pulse methylprednisolone in the ICU and had a concomitant probable pulmonary aspergillosis and *Klebsiella pneumoniae* pneumonia). One patient had both IA (*Aspergillus fumigatus*) and severe mucormycosis (*Rhizopus arrhizus*) (Supplementary Figure 3). All of these patients received azole therapy and 2 died, 81 and 125 days after IA diagnosis (Table 2).

Eight patients with SARS-CoV-2 persistent viral shedding died. Five had hematological malignancies, 2 were KTRs, and 1 had a combined immunodeficiency (Table 2). Five were hospitalized in the ICU: 4 of them presented documented *Pseudomonas aeruginosa* pneumonia and 3 had IA (2 probable and 1 possible). For 7 of these patients, death was related to complications of COVID-19, bacterial superinfections, or AI, and 1 patient died from progression of his hematological malignancy (Table 3).

### Risk Factors for Persistent Viral Shedding

Baseline characteristics were similar in both groups regarding age, sex, and comorbidities (Table 1). In univariate analysis, the 2 groups of patients received comparable immunosuppressive drugs (Table 1). Non–anti-SARS-CoV-2 vaccinated patients were significantly more represented in the group of persistent viral shedding. Patients with persistent viral shedding presented significantly more profound lymphopenia (<0.5 G/L) and were more frequently infected with the Alpha and Omicron variants (χ^2^ test, *P* < .05) (Table 1). We did not identify any significant difference regarding documented bacterial infections between the 2 groups. In the multivariate analysis, non–anti-SARS-CoV-2 vaccinated patients had a significantly 6-fold increased risk of persistent viral shedding (>8 weeks) (odds ratio [OR]: 6.6; 95% confidence interval [CI], 1.73–25.1; *P* = .006). Fever and lymphopenia (<0.5 G/L) at diagnosis were significantly associated with a 4- and 3-fold increased risk of persistent viral shedding, respectively (OR: 3.3; 95% CI, 1.01–11.09; *P* = .048; OR: 4.3; 95% CI, 1.2–14.7; *P* = .019).

### Complications of Persistent Viral Shedding (>8 Weeks)

**Figure 2.**
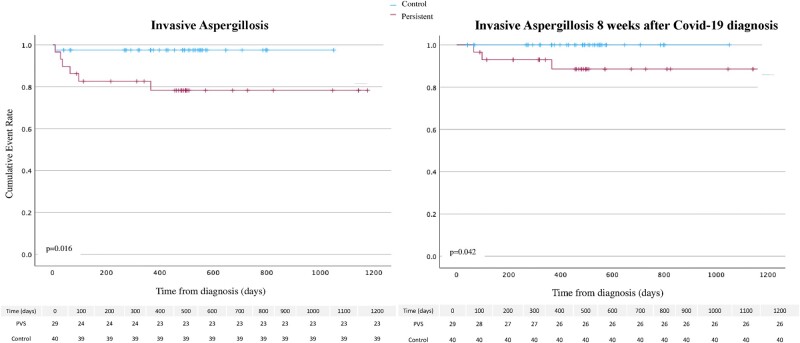
Invasive aspergillosis, Kaplan-Meier (*P* < .001).

The group of patients with persistent viral shedding (>8 weeks) had a 4.8-fold increased risk of hospitalization (OR: 4.8; 95% CI, 1.5–15.6; *P* = .008) and a 10-fold increased risk of IA (OR: 10.17; 95% CI, 1.15–89.8; *P* = .037). In a Kaplan-Meier analysis, patients with persistent viral shedding (>8 weeks) were significantly more at risk of death and more at risk of developing IA (log-rank test <0.05) (Figure 2 and Supplementary Figure 4). The use of plasmatherapy was significantly associated with persistent viral shedding (OR: 17.2; 95% CI, 1.9–152.1; *P* = .01).

## DISCUSSION

In the present study, we identified an emerging group of patients with dramatic persistent SARS-CoV-2 viral shedding who presented an approximate 5-fold increased risk of hospitalization, a 10-fold increased risk for IA, and a significantly increased risk for death, highlighting the potential disease severity in IC patients.

We report a large series of IC patients with persistent viral shedding, including 16 SOT patients (mostly KTRs). To the best of our knowledge, 88 IC patients with persistent viral shedding have been reported in 70 carefully analyzed articles in the literature. Most had hematological malignancies (n = 65) and a few other conditions (Supplementary Figure 5; Supplementary Table 2). According to these 88 cases, persistent viral shedding was reported with a mean viral shedding that was slightly longer than what we observed in our cohort. Sixteen of the patients reported in the literature died (18%) and 8 (9%) had IA, which is less than what we observed here (27% and 20.6%, respectively) [13, 28–34]. Recently, virus-associated pulmonary aspergillosis (influenzae- and COVID-19–associated pulmonary aspergillosis) confirmed the invasiveness of aspergillosis in a series of autopsies carried out on patients who had been hospitalized in the ICU [26]. Proven invasive pulmonary aspergillosis diagnosis presented a similar histological pattern in influenza and in COVID-19 ICU case fatalities, highlighting the need to consider this invasive fungal infections in patients with COVID-19 [26]. Invasive aspergillosis can occur in the setting of viral infections because of virus-induced alteration of the respiratory epithelium and modification of immune responses, a phenomenon accentuated by mechanical ventilation and corticosteroid therapy in the ICU [35, 36]. In immunocompromised patients not hospitalized in the ICU, this mechanism is supported by the absence of cellular and humoral responses against both agents, virus and fungus [37]. Nevertheless, the exact pathophysiology leading to invasive aspergillosis in non-ICU immunocompromised patients infected with SARS-CoV-2 has not been clearly identified.

Several SARS-CoV-2 mutations in IC patients were described in the literature; some were involved in viral resistance to remdesivir, nirmatrelvir/ritonavir, and monoclonal antibodies, explaining the persistence of the viral shedding in those patients [18, 20, 38–43]. In a recent study of 150 IC patients with Omicron SARS-CoV-2 infection, only 9 patients had persistent viral shedding for more than 8 weeks [16].

According to the literature, we identified that vaccination was protective against persistent viral shedding in IC patients [44]. Unvaccinated patients had a significant 6-fold increased risk of persistent viral shedding. In the literature, 6%–69% of the SOT patients reached serological response with a third dose of vaccine. Thus, 24% of KTRs remained seronegative after a third dose of vaccine, and a lower neutralization immune response against evasive variants was observed in IC patients compared with immunocompetent patients after receiving a booster [45–48]. Although implementing preventive measures (vaccination and monoclonal antibodies) may be insufficient in this group of patients, the therapeutic challenge has not yet been resolved.

We observed that persistent viral shedding was significantly associated with plasma therapy and combined therapy (monoclonal antibodies and plasma therapy). Some authors reported the efficacy of a prolonged use of nirmatrelvir/ritonavir for more than 20 days in association with remdesivir for more than 19 days after failure of monoclonal antibodies, intravenous remdesivir, and prolonged oral corticosteroids, which were not used here [49]. Recently, Mikulska et al proposed combination therapy with 2 antivirals and monoclonal antibodies in symptomatic IC patients with Omicron persistent viral shedding [44]. They showed that the 14- and 30-day responses were significantly better when combination therapy included monoclonal antibodies [44]. According to the literature review, most patients received combined therapies associating remdesivir, monoclonal antibodies, and convalescent plasma, without systematic viral clearance. To date, no standardized recommendations are available for IC patients with persistent viral shedding.

Our study has some limits. We likely underestimated the number of patients with persistent viral shedding because some patients had no control nasopharyngeal PCR, others died with a positive SARS-CoV-2 infection without control, and because we lacked clinical data for most of them. We did not observe a significant association between persistent viral shedding, B-cell depletion, and the use of anti-CD20 or CD19 antibodies, although it was reported in the literature [19, 38]. This could be linked to a lack of power resulting from the small number of patients suffering from hematological disorders and treated with B-cell–depleting therapies, as well as the high proportion of SOTs who did not receive anti-B therapies.

In conclusion, our study showed that vaccine coverage against SARS-CoV-2 was protective against persistent viral shedding in IC patients. Immunocompromised patients with persistent viral shedding were at risk for IA and death. This population should therefore be systematically screened for IA using galactomannan antigen and followed up with systematic CT scans to identify lung lesions suggestive of IA and perform bronchoalveolar lavage to confirm the diagnosis.

## Supplementary Material

ofae012_Supplementary_Data

## Supplementary Data

Supplementary materials are available at *Open Forum Infectious Diseases* online. Consisting of data provided by the authors to benefit the reader, the posted materials are not copyedited and are the sole responsibility of the authors, so questions or comments should be addressed to the corresponding author.
