# Supplementary material for: Increased Risk of Invasive Aspergillosis in Immunocompromised Patients With Persistent SARS-CoV-2 Viral Shedding >8 Weeks, Retrospective Case-control Study
Source: Open Forum Infect Dis. 2024 Jan 9;11(2):ofae012. doi: 10.1093/ofid/ofae012 (PMC10883287; doi:10.1093/ofid/ofae012)
Supplement: ofae012_Supplementary_Data [file ofae012_supplementary_data.docx]

**SUPPLEMENTARY DATA**

**SUPPLEMENTARY DATA**

**Supplementary Figure 1.** **Medical history of patients with persistent SARS-CoV-2 viral shedding > 8 weeks and controls.**

1. Immunocompromised patients with persistent viral shedding >8 weeks
2. Immunocompromised patients with persistent viral shedding >8 weeks
3. Immunocompromised patients with viral shedding ≤ 8 weeks
4. Immunocompromised patients with viral shedding ≤ 8 weeks

SOT: solid organ transplant; HIV: Human Immunodeficiency Virus; ANCA: Anti Neutrophils Cytoplasmic Antibody; COPD: Chronic obstructive pulmonary disease; DLBCL: Diffuse and Large B-cell Lymphoma; IEI: Inborn Error Immunity

**Supplementary Figure 2.** **Persistent viral shedding in days according to the type of immunosuppression in patients with persistent viral shedding (>8 weeks).**

**Supplementary Figure 3.** **Invasive aspergillosis and mucormycosis in an immunocompromised patient with persistent viral shedding.**

**Supplementary Figure 4.** Kaplan Meier analysis of death in IC patients with SARS-CoV-2 infection

**Supplementary Figure 5.** Medical history of persistent viral shedding among immunocompromised patients reported in the literature (n=80).

**Supplementary Table 1.** Documented bacterial infections in patients with persistent viral shedding.

**Supplementary Table 2.** Persistent SARS-CoV-2 viral shedding in immunocompromised adults: review of the literature.

**Supplementary Figure 1.** **Medical history of patients with persistent SARS-CoV-2 viral shedding > 8 weeks and controls.**

1. Immunocompromised patients with persistent viral shedding >8 weeks
2. Immunocompromised patients with persistent viral shedding >8 weeks
3. Immunocompromised patients with viral shedding ≤ 8 weeks
4. Immunocompromised patients with viral shedding ≤ 8 weeks

SOT: solid organ transplant; HIV: Human Immunodeficiency Virus; ANCA: Anti Neutrophils Cytoplasmic Antibody; COPD: Chronic obstructive pulmonary disease; DLBCL: Diffuse and Large B-cell Lymphoma; IEI: Inborn Error Immunity

**Supplementary Figure 2.** **Persistent viral shedding in days according** **to the type of immunosuppression in patients with persistent viral shedding (>8 weeks).**

Abbreviations: COPD: chronic obstructive pulmonary disease, HIV: human immunodeficiency virus, SOT: solid organ recipient

**Supplementary Figure 3.** **Invasive aspergillosis and mucormycosis in an immunocompromised patient with persistent viral shedding.**

A-B. Lung CT scan at 346 days post-infection with positive SARS-CoV-2 on nasophayngeal sample (Ct value 20,51).

C. PET-TDM at 417 post-SARS-CoV-2 infection. D. Sinus CT-scan with filling of the right maxillary sinus and lysis of the lateral wall of the extended sinus (arrowhead). A 55-year-old lung transplant recipient women treated with Tacrolimus, steroids, and MMF, was diagnosed with probable sinusal mucormycosis and invasive pulmonary aspergillosis. BAL culture was positive for *A. fumigatus*,Galactomannan antigen was positive at 2,76 and blood PCR was positive for *A. fumigatus* 560 copies per mL. Culture of the sinusal biopsy was positive for *Rhizopus arrhizus,* and histology showed invasive filamentous infection.

**Supplementary Figure 4.** Kaplan Meier analysis of death in IC patients with SARS-CoV-2 infection

**Supplementary Figure 5.** Medical history of persistent viral shedding among immunocompromised patients reported in the literature (n=80).

Abbreviations: HIV: human immunodeficiency virus; IEI: Inborn Error Immunity; SOT: solid organ transplantation.

**Supplementary Table 1. Documented bacterial infections in patients with persistent viral shedding.**

| **Number of documented bacterial infection** | **Site of infection** | **Bacterial documentation** | **Treatment** |
| --- | --- | --- | --- |
| 1 | Sinusitis | MSSA | Amoxicillin-clavulanate |
| 2 | Urinary tract infection | *E. faecium* | ofloxacin |
| 3 | Pneumonia | *P. aeruginosa* | Ceftazidim-Meropenem |
| 4 | Pneumonia  Bacteriemia | *P. aeruginosa*  *K. pneumoniae* | Ciprofloxacin  Imipenem |
| 5 | Pneumonia | *K. pneumoniae* | Piperacillin-Tazobactam |
| 6 | Pneumonia | *P. aeruginosa* | Ceftazidim |
| 7 | Digestive  Pneumonia  Bacteremia | *C. difficile*  *H. influenzae*  *E. cloacae* | Fidaxomicin  Cefepime |

MSSA: methicillin sensitive S. aureus

**Supplementary Table 2. Persistent SARS-CoV-2 viral shedding in immunocompromised adults: review of the literature**

| Patient | Variant | Year | Sex/Age (years) | Immunodepression | IS drugs | VS duration (Days) | Clinical manifestations | Treatment | Relapses | IA | Outcome | SARS-CoV-2 mutations | Mutation significance | References |
| --- | --- | --- | --- | --- | --- | --- | --- | --- | --- | --- | --- | --- | --- | --- |
| 1 | NA | 2020 | M/45 | APLS | CTC, cyclophosphamide  R, eculizumab  IVIg,  ruxolitinib | 154 | Fever, abdominal pain and dyspnea, | Remdesivir | Yes | Yes | Died | Orf1a  RdRp  Spike | Spike and RBD | Choi, *NEJM*, 2020 [1] |
| 2 | Wuhan | 2020 | F/76 | CLL | R/Bendamustine | 21 | Cough**,** fever, dyspnea, hypoxaemia | Remdesivir  CPT | No | No | Cured | RdRP mutation (D484Y) | Remdesivir mutation | Martinot, *Clin Infect Dis*, 2021[2] |
| 3 | A.1 | 2020 | F/71 | CLL | No | 70 | Asymptomatic | 2 CPT | No | No | NA | Gene Orf1ab ; Spike ; M ; Orf8 ; N | NTD ; S1 S | Avanzato, *Cell*, 2020 [3] |
| 4 | NA | 2020 | M/47 | FL | Binutuzumab,bendamustine | 60 | Asymptomatic | Favipiravir, ciclesonide  lopinavir/ritonavir | Yes | No | Cured | NA | NA | Nakajima, *J of Infection and Chemotherapy,* 2021[4] |
| 5 | Wuhan | 2020 | M/49 | Kidney Transplant | Tacrolimus  MMF  CTC | 53 | Fever, fatigue | umifenovir  Stop IS drugs CTC | Yes | No | Cured | NA | NA | Man, *Am J Transplant*, 2020[5] |
| 6 | B.1 lineage and 20A | 2020 | M/57 | Eosinophilic granulomatosis with polyangiitis | MMF  CTC,  R | 210 | Fever, dry cough, anosmia, headache | Remdesivir  CPT | No | No | Cured | Q675H substitution  M85-V86 deletion in ORF1a  The R2613N  Q1140K  P2612L  D138E  H146N  G446V  N679K | Spike  (NTD) (RBD)  *in vitro*  decrease sensitivity to CPT and monoclonal antibody REGN10987  Furin cleavage | Spinicci, *J Clin Immunol*, 2022[6] |
| 7 | 20.B | 2020 | M/70 | Marginal BCL | vincristine, CTC, CPMD ; R | 66 | NA | Remdesivir  CPT | NA | No | Died | D614G Spike variant  I513T in NSP2 (T2343C) and V157L (G13936T) in RdRp  spike variant N501Y,  D796H+  ΔH69/ΔV70_ | Increase the ACE2 receptor affinity  Spike mutants | Kemp, *Nature*, 2021[7] |
| 8 | 20B, lineage B.1.1. | 2020 | F/52 | FL | CPMD , vincristine, doxorubicin, CTC, obinutuzumab | 95 | Fever, diarrhea, and fatigue | None | No | No | Cured | 11 point mutations | 3 AA substitutions in the gene coding for the S protein (S:S50L, S:A653V, and S:L1186F) | Lynch, *Emerging Infect Dis*, 2021[8] |
| 9 | Wuhan-Hu-1 | 2020 | M/60 | B-cell lymphoma | CPMD, doxorubicin, CTC | 119 | Epistaxis ; productive cough | Remdesivir  CPT | 3 clinical relapses | No | Cured | ORF1b M101L | remdesivir resistance | Baang, *J Infect Dis*, 2021[9] |
| 10 | Pangolin B.1.1 20B | 2020 | M/70 | Mantle lymphoma | R, bendamustine, cytarabine | 240 | ARDS, ICU, CPAP | Darunavir/ritonavir  Remdesivir  CPT | 4 clinical relapses | No | NA | ORF1A  ORF1B  Spike | NA | Sepulcri, *Open Forum Infectious Diseases*, 2021[10] |
| 11 | Pangolin lineage B.1.1  Nextstrain clade 20B | 2020 | F/60 | CLL | R; Bendamustine | 210 | Fever, cough, headache pain, fatigue, cough | CPT | Yes | No | Cured | 17 mutations in Spike  15 VOC/VOI  (S:E484K, S:D950N, S:P681H, S:N501Y, S:del(9), N:S235F and S:H655Y)  ORF1a  ORF3a | immune escape mutations | Sonnleitner, *Nature Comm*, 2022[11] |
| 12 | Wuhan | 2020 | M/66 | HIV (CD4/0 and JC encephalopathy) | No | 111 | Asymptomatic  CT scan increased Covid 19 lesions | NA | No | No | NA | C23718T (transient)  14 mutations differed from Wuhan strain | NA | Tarhini, *J Infect Dis*, 2021[12] |
| 14 | NA | 2021 | M/35 | Rheumatoid arthritis | R | 73 | Cough; dyspnea fever | CTC | Yes | No | NA | C5147T mutation | Possible 2 strains | Tarhini, *J Infect Dis*, 2021[12] |
| 13 | NA | 2021 | F/37 | FL | R, etoposide, cisplatin, cytarabine, CTC | 60 | Fever, dyspnea | lopinavir/ritonavir, hydroxychloroquine azithromycin  CTC  anakinra  Remdesivir | Yes | No | Cured | NA | NA | Camprubi, *Int J infect Dis*, 2021[13] |
| 14 | Pango B1  20C | 2022 | F/70 | NHL | R -bendamustine | 164 | Fever, anosmia, cough and rhinorrhea, refractory anemia and neutropenia | Casirivimab/imdevimab | No | No | Cured | E802D, in the nsp12 RNA-dependent RNA polymerase | *In vitro* Remdesivir resistance | Gandhi, *Nature Communication*, 2022[14] |
| 15 | Alpha B.1.1.7 | 2022 | F/84 | Mantle cell lymphoma | R- lenalidomide | 234 | No respiratory manifestation/ground glass opacities | CTC | No | No | NA | ORF1a  Spike p  Matrix p  Enveloppe p  ORF3a  ORF7 | Immune evasion | Fourati, *Em Infect Dis*, 2022 [15] |
| 16 | Omicron/BA.1 | 2022 | M/47 | HI(CD4+ ::47/mm3) | No | 39 | Dyspnea cough concomitant pneumocystis infection | Sotrovimab | No | No | Cured | NA | NA | Adachi, *Internal med*, 2022 [16] |
| 17 | Omicron | 2022 | F/70 | Large B-cell lymphoma | R | 72 | Dyspnea | Remdesivir  ICU  sotrovimab | No | No | Cured | NA | NA | Wee, *IDcases*, 2022 [17] |
| 18 | Omicron | 2022 | F/42 | Systemic sclerosis | R-CTC | 64 | Fever ; dyspnea ; fatigue | nirmatrelvir/ritonavir; tixagevimab/cilgavimab | No | No | Cured | NA | NA | Wee, *IDcases*, 2022[17] |
| 19 | Omicron | 2022 | M/68 | FL (CD4 : 50/mm3) | obinutuzumab | 270 | Fever  hypoxaemia | Remdesivir  Casirivimab/imdevimab | No | No | Cured | NA | NA | Nagai, *Jpn J Infect Dis.* 2022[18] |
| 20 | Pangolin [lineage](https://www.sciencedirect.com/topics/immunology-and-microbiology/lineages) B.1.1.306 | 2022 | F/21 | ALL | ASCT  [blinatumomab](https://www.sciencedirect.com/topics/medicine-and-dentistry/blinatumomab)  inotuzumab | 270 | Worsening symptoms 2 weeks after inotuzumab | CTC  Remdesivir  Voriconazole (CAPA) | No | Yes | Death | 7 mutations,  6 protein changes  ORF1ab,  S & N | Unknown | Leung, *International J of Infectious Diseases*, 2022[19] |
| 21 | NA | NA | F/5months | SCID  (mutation in*IL7R* gene) | NA | 180 | MIS  chronic oral candidiasis, protracted diarrhea, mycobacterial infection | IvIg ,anticoagulants | No | No | NA | NA | NA | Hariharan, *Indian J Pediatrics,* 2022 [20] |
| 22 | BA.1.1 | 2020 | M/45 | NHL | Chemotherapy | 102 | Fever cough  ARDS | Remdesivir  CPT | Yes | No | Died | 9 AA substitutions in five viral genes (Nucleocapsid, ORF1a, ORF1b, ORF13a, and ORF9b | NA | Villasenor-Echevarri, *Viruses*, 2023 [21] |
| 23 | NA | NA | M/72 | FL | R-CHOP | 124 | Cough, fever and dyspnea | nirmatrelvir/ritonavir | Yes | No | Cured | NA | NA | Lindahl, *Infect Dis*, 2023[22] |
| 24 | NA | NA | M/12 | Immune thrombocytopenia | CTC | 98 | Cough and fever |  | Yes | Yes | NA | NA | NA | Reddy et al, *Cureus*, 2023[23] |
| 25 | NA | 2020 | M/75 | CLL | R  cyclophosphamide  bendamustine  Ibrutinib | 333 | Fever and dyspnea | Remdesivir  CPT | Yes | No | Cured | deletion of amino acids Y144 and LLA241-243 in the NTD, and a F490S mutation in the RBD  ORF1b:D31Y) | enhanced transmission properties and increased resistance towards convalescent plasma and vaccinee sera  increased resistance to neutralizing monoclonal antibodies | Monrad, *OFID*, 2022[24] |
| 26 | NA | 2022 | M/64 | CLL | venetoclax and obinutuzumab | 120 | Fever, cough, dyspnea, COPB | nirmatrelvir/ritonavir  Remdesivir  Methylprednisone | Yes | No | Cured | NA | NA | Trottier, *Clin infect Dis*, 2022[25] |
| 28 | NA | 2020 | F/NA | DLBCL | R-  Chemotherapy | 318 | Fever, pneumonia | CPT | Yes | No | NA | 40 changes | HLA class I antigen  Loss CD8-T cell response | Stanevich, *Nat Comm*, 2023[26] |
| 29 | NA | NA | M/21 | ALL | CD19-CAR-T | 250 | Fever and dyspnea | Remdesivir  CPT | NA | No | Cured | NA | NA | Truong, *EbioMedicine*, 2021[27] |
| 30 | B1.1.7 (alpha VOC), | NA | M/71 | CLL | FCR chemotherapy | 305 | NA | Remdesivir (5 days, D213-217) IV immunoglobulin (D216) Casirivimab/ imdevimab (D266) | NA | No | Cured | ΔH69/ΔV70 al  H655Y | Spike protein mutation | Hettle, *Clin Infect Pract,* 2022[28] |
| 31 | NA | NA | F/53 | AML | Ongoing ciclosporin + Sorafenib Bone marrow transplant | 154d | NA | Remdesivir | NA | No | Died | T205I  M86V | NA | Hettle, *Clin Infect Pract*, 2022[28] |
| 32 | NA | NA | F/58 | NHL | Obinutuzumab | 269 | NA | Remdesivir Casirivimab/ imdevimab | NA | No | Died | E420D | NA | Hettle, *Clin Infect Pract*, 2022[28] |
| 33 | NA | NA | F/80 | CLL | Previous Ibrutinib | 94 | NA | None | NA | No | Died | E484K | NA | Hettle, *Clin Infect Pract*, 2022[28] |
| 34 | NA | NA | M/42 | ALL | Bone marrow transplant - haploidentical allograft | 58 | NA | Remdesivir | NA | No | Died | E484K | NA | Hettle, *Clin Infect Pract*, 2022[28] |
| 34 | NA | NA | F/69 | DLBCL | R-CHOP chemotherapy | 113 | NA | Tocilizumab | NA | No | Died | NA | NA | Hettle, *Clin Infect Pract*, 2022[28] |
| 36 | BA.2 | NA | M/80 | FL | Chemotherapy | 49 | Fever and Dyspnea | tixagevimab/cilgavimab  CPT  nirmatrelvir/ritonavir | Yes | No | Cured | NA | NA | Blennow, *Clin Infect Dis*, 2023[29] |
| 37 | NA | 2020 | M/23 | ALL | R  HCST | 404 | Dyspnea | CPT  Remdesivir  REGN-COV2 | No | No | Cured | N501Y mutation | NA | Bailly, *Clin Infect Dis*, 2022[30] |
| 38 | BA.5.2.3 | 2022 | M/73 | FL | RBendamustine  Lenalidomide | NA | Cough, fever, rhinorrhea | cilgavimab/tixagevimab molnupiravir and nirmatrelvir/ritonavir | Yes | No | Cured | L50F, E166V, L167F) | NA | Marangoni, *Int J Infect Dis*, 2023 |
| 39 | NA | NA | F/63 | Lymphoma | Tirabrutinib  ASCT | 189 | Fever, Sore throat | Tocilizumab, mAb | Yes | No | Died | NA | NA | Nagasaki, *Medicina*, 2023[31] |
| 40 | BA.1.1. | 2022 | M/56 | NHL | R  Bendamustine | 45 | Fever, mild respiratory distress syndrome | Sotrovimab  Molnupiravir  Remdesivir  Dexamethasone  nirmatrelvir/ritonavir  Sotrovimab | Yes | No | Died | NA | NA | Rizzo, *J Med Virol*, 2023[32] |
| 41 | BA.1.1. |  | M/65 | Lymphoma | R | 196 | Fever, sore throat, dyspnea | sotrovimab, remdesivirnirmatrelvir/ritonavir, and molnupiravir. | Yes | Yes | Lung interstitial fibrosis | (3 chymotrypsin-like protease [3CLpro] E166 A/V),  spike P337L and E340K)  RNA-dependent RNA polymerase [RdRp] V166L | resistance mutations to nirmatrelvir  sotrovimab  remdesivir | Hirostu, *Med*, 2023[33] |
| 42 | Alpha | NA | M/76 | HIV (CD4 : 4/mm3) | None | 142 | Fever, cough, dysphagia | None | Yes | No | Died | None | None | Zhabokristky, *Infect Control Host Epidemiol*, 2023[34] |
| 43 | B.1.617.2 AY.122 lineage | 2021 | M/63 | FL | RBendamustine | 210 | Dyspnea | Bamlanivimab/Etesevimab  Methylprednisolone  nirmatrelvir/ritonavir | No | No | Cured | ORF1ab  S gene  Spike N-Terminal Domain  Recurrent Deletion Regions | escape to mAb  enhance binding affinity to the ACE2 | Brandolini, *Viruses*, 2023[35] |
| 44 | NA | NA | M/36 | HIV ( CD4^+^ : 5/mm^3°)^ | None | 270 | Cough, weight loss | None | No | Yes | NA | NA | NA | Mhanna,[36] |
| 45 | NA | NA | M/30 | HIV (CD4 : 49/mm3)/DLBCL | Anti CD20 mAb  polatuzumab vedotin, Rand bendamustine | 97 | Fever | Remdevisir  CPT  mAb | No | No | Cured | NA | NA | Montejano, *AIDS*, 2022[37] |
| 46 | NA | 2020 | M/74 | Kidney transplant | R  Tacrolimus  MMF  CTC | 90 | Dyspnea and fever | None | Yes | No | Cured | G142D mutation  deletion of 3-amino-acids at position 143-145 | NA | Morel, *Am J Transplant*, 2022[38] |
| 47 | Delta | 2021 | M/53 | AML | ASCT  ruxolitinib | 94 | Fever, dyspnea, cough | Remdesivir | Yes | No | NA | 445, 446, 476, 477, and 493 of the spike protein | broad resistance to monoclonal antibody panels | Ko, *Microbiol Spectr*, 2022[39] |
| 48 | Delta | 2021 | M/67 | Splenic Marginal lymphoma | R | 97 | Cough and dyspnea | Remdesivir  DXM  REGN-COV2  tocilizumab | No | Yes | NA | 445, 446, 476, 477, and 493 of the spike protein | broad resistance to monoclonal antibody panels | Ko, *Microbiol Spectr*, 2022[39] |
| 49 | Omicron |  | M/71 | FL | Anti-CD20 Bendamustine | >8 weeks | Fever | Remdesivir | No | No | Cured | NA | NA | Arai, *J Infect Chemother*, 2022[40] |
| 50 | B.1.160 by Pangolin | 2020 | M/61 | ClL | fludarabine, cyclophosphamide and R | 348 | Fever, nausea, dry cough  Weight loss | Remdesivir  CPT | Yes | No | Cured | NA | NA | Schenker, *Br J Hemarol*, 2022[41] |
| 51 | B.1.1.7 (Alpha) | N1 | M/22 | Granulomatous–lymphocytic interstitial lung disease | Azathioprine  R | 77 | Fever dyspnea | Remdesivir CPT  DXM | Yes | No | Cured | NA | NA | Navalon, *Clin Microbiol Infect*, 2022[42] |
| 52 | Wuhan | 2020 | M/51 | NHL | R | 133 | Fever, dyspnea, hypoxia | Remdesivir | Yes | No | BOOP  Cured | NA | NA | Morishita, *J Infect Chemother*, 2022[43] |
| 53 | NA | NA | F/39 | Lupus | Anti-CD20 m Ab | 94 | Hypoxaemia | CPT | Yes | No | Cured | NA | NA | Moutinho-Pereira, *BMJ Case report*, 2021[44] |
| 54 | NA | NA | M/55 | FL | R CHOP  R maintenance | 200 | Fever | REGN-COV2, Ronapreve  Remdesivir | No | No | Cured | D614G and T723I  two additional spike protein changes  deletion of 141–143 i | NA | Taha, Ann Clin Microbiol Antimicrob, 2021[45] |
| 55 | NA | NA | F/68 | CLL | Ibrutinib | 290 | Fever hypoxaemia | Remdesivir  REGN-COV2 | Yes | No | Cured | NA | NA | Taha, *Ann Clin Microbiol Antimicrob*, 2021[45] |
| 56 | B.1.332  Alpha | 2020 | F/48 | B-cell lymphoma | Anti-CD20  CAR-T cells | 335 | Fever, headache, cough | Remdesivir  CPT | No | No | COPD  Cured | ORF7b and ORF8 | NA | Nussenblatt, *J Infect Dis*, 2022[46] |
| 57 | NA | NA | M/59 | FL | Anti-CD20  R-bendamustine  Obinutuzumab  bendamustine | 312 | Fever cough and dyspnea | REGEN-COV (10933 and 10987)  mAb  lopinavir/ritonavir  CPT  Remdesivir | No | No | Cured | AA mutation N and S | NA | Drouin, Viruses, 2021[47] |
| 58 | NA | 2020 | F/25 | SCID RAG1 variant | HSCT | 56 | Pulmonary infection | Remdesivir  CPT | No | No | Cured | NA | NA | Keitel, *Front Immunol*, 2021[48] |
| 59 | NA | NA | F/63 | FL | obinutuzumab and bendamustine | 54 | Pulmonary symptoms | Remdesivir | No | No | Cured | NA | NA | Ueda, *Internal Medicine*, 2022[49] |
| 60 | B.1.1.214 | 2020 | M/61 | FL | R and bendamustine | 100 | Fever | Favipiravir  Remdesivir  IvIg | No | No | Cured | E484Q and S494P  ORF1b and ORF9b  V658I, was located in nsp12 | Spike protein  RBD | Shoji, *J infect Chemother*, [50] |
| 61 | B.1.1.7 | 2021 | M/68 | CLL | fludarabine, cyclophosphamide, and R  venetoclax | 90 | Pneumonia | Remdesivir  CPT | No | No | Cured | Glu484Gln | spike gene mutations | Bronstein, *J Med* *Virol*, 2022[51] |
| 62 | B.1.362 | 2021 | M/33 | HL | dacarbazine, doxorubicin, and vincristine | 56 | Dyspnea | NA | No | NA | NA | NA | spike mutations | Bronstein, *J Med Virol*, 2022[51] |
| 63 | B.1.1.209 | 2020 | M/77 | CLL | R, Fludarabine and cyclophosphamide and  ibrutinib | 64 | Dyspnea and hypoxaemia | Remdesivir  CPT | No | Yes | Cured | T820I and P822L amino acid-replacements located in NSP3 | NA | Hanssen, *Infect Dis Repo*, 2021 [52] |
| 64 | B.1.1 | 2020 | M/59 | FL | CHOP | 222 | Dyspnea | None | No | No | Cured | Q493K and N501T | Fusion at the plasma membrane (PM)  increased production of interferons (IFNs)  65escape the antiviral activity | Caccuri, *Virus Evol*, 2022[53] |
| 66 | B.1.1.7 | 2020 | F/61 | DLBCL | R | 180 | Pneumonia | Remdesivir | No | No | Fibrotic changes | four amino acid changes (V3G, S50L, N87S, and A222V) and two deletions (18-30del and 141-144del) | Spike protein  convergent adaptive evolution | Borges, *mSphere*, 2022[54] |
| 67 | Delta AY.100 | 2021 | NA/45 | HIV (CD4+ : 2/mm3) | None | >80 | Fever, chills, nonproductive cough, dyspnea | CPT | No | No | Cured | V3G, L18F, H245P, and E484K | Spike RBD | Peters, *OFID*, 2022[55] |
| 68 | B.1.2 | 2020 | F/65 | CLL | PSCT  ASCT  tacrolimus and R | 302 | Nausea, vomiting, cough, and sore throat | Remdesivir | Yes | No | Cured | Q677P | Spike protein | Scherer, *NEJM*, 2022  [56] |
| 69 | B.1.2 | 2020 | F/46 | DLBCL | Anti CD20  R CHOP | 75 | Dyspnea, fever | Remdesivir | Yes | No | Died | Q493R | Spike protein evolution | Scherer, *NEJM*, 2022  [56] |
| 70 | B.1.2 | 2020 | F/38 | MDS | PBSCT  GVHD  R  MMF  CTC | 109 | Dyspnea, fever, cough | Remdesivir | NA | No | NA | E484Q  V143- Y144 | Spike protein evolution | Scherer, *NEJM*, 2022  [56] |
| 71 | B.1.568 | 2020 | M/40 | Thymoma | Thymectomy | 200 | Dyspnea, fever, cough | Remdseivir  CPT | Yes | No | Cured | NA | NA | Scherer, *NEJM* 2022  [56] |
| 72 | B.1.493 |  | M/46 | MZL | R bendamustine | 302 | Dyspnea | Remdesivir  mAb | NA | No | Cured | NA | Spike protein evolution | Scherer, *NEJM*, 2022  [56] |
| 73 | NA | 2020 | F/74 | CLL | R bendamustin | 78 | Asthenia, dry cough, weight loss | CPT | NA | No | Cured | NA | NA | Zimmerli, *Front Immunol,* 2021[57] |
| 74 | B/19A | 2020 | M/52 | FL | R | 188 | Fever | Lopinavir/ritonavir  Remdesivir | Yes | Yes | Died | reading frame (ORF) *1ab* (four), *ORF 7a* (one), and *ORF 8a* | NA | Perez-Lago, *Biomedicine*, 2021 [58] |
| 75 | B.1.5/20A | 2020 | M/42 | FL | R-bendamustine | 141 | Fever respiratory failure | lopinavir/ritonavir  tocilizumab  anakinra  CPT | NA | No | Cured | ORF1ab (seven), ORF 8 (one), S (two), and N (two) | NA | Perez-Lago, *Biomedicine*, 2021[58] |
| 76 | A.5/19B | 2020 | M/63 | FL | R bendamustine | 69 | Fever and pancytopenia. | Remdesivir lopinavir/ritonavir treatment  CPT | No | No | Died | ORF1ab | NA | Perez-Lago, *Biomedicine*, 2021[58] |
| 77 | B.1.1.29 | 2020 | F/70 | FL | R CHOP  obinutuzumab | 156 | ARDS | CPT  Remdesivir | NO | No | Cured | D614G  144 NTD  S477N  E484K  Non spike protein  ORF1ab T4164I | Spike protein : Evasion of polyclonal Ab  Elimination CD8T-cell recognition | Khatamzas, *Nature Comm*, 2022[59] |
| 78 | NA | 2020 | F/61 | FL | R | 365 | Pneumonia | None | Yes | No | Cured | NA | NA | Yasuda, *Clin lymphoma Myeloid Leuk*, 2021[60] |
| 79 | PANGO lineage A.2 (Clade 19B) | 2020 | F/23 | X-linked-Agammaglobulinemia |  | 149 | Bilobar pneumonia, reporting cough, chronic diarrhea, and fever | Remdesivir, lopinavir/ritonavir  CPT | No | No | Died | ORF1a:F3701Y, ORF3a:G196V, ORF8:L84S, N:S197L  I197V  A653V | S gene  escape mutation against anti-SARS-CoV-2 neutralizing antibodies | Ciuffreda, *J Infect* 2021[61] |
| 80 | PANGO B.1 lineage | 2020 | M/58 | FL | R  bamlanivimab | 189 | Cough, low-grade fever, and general malaise | Remdesivir | Yes | No | Cured | NA | NA | Thornton, Antimicroboal Resistance Infect Control, 2022[62] |
| 81 |  | 2020 | M/71 | Mantle cell lymphoma | R; cyclophosphamide, doxorubicin, vincristine, and CTC | 164 | Cough, fever, dyspnea | Favipiravir  Remdesivir  CPT | Yes | No | Cured | NA | NA | Berkthas, *Am J Trop Med Hyg*, 2022[63] |
| 82 | Wuhan | 2020 | F/69 | Heart transplant  grade 1B acute cellular rejection SOT | CTC, tacrolimus, MMF, | 134 | Hypoxaemia | Remdesivir  Tocilizumab | Yes | No | Cured | D138Y | S gene | Purpura, *Am J Transplant*, 2022[64] |
| 83 | PANGO lineage B.1.2 | 2020 | F/46 | Multiple sclerosis | ocrelizumab | 70 | Fever and dyspnea | Remdesivir  CPT | Yes | No | Cured | 19 missense mutations | Unknown | Gibson, *OFID*, 2022[65] |
| 84 | NA | 2021 | M/73 | MM | HSCT  CAR T-cell therapy after fludarabine/cyclophosphamide lymphodepletion | 72 | Fever | Tocilizumab Remdesivir CPT | Yes | No | Died | Y144 deletion and a D215G  N501T substitution | enhance binding affinity to the ACE2 receptor | Hensley, *Clin Infect Dis*, 2021[66] |
| 85 | B.1.1.7  Alpha | 2021 | M/40 | HIV | None | 111 | Dyspnea | None | No | No | Cured | mutations in the RBD | NA | Riddell, *Clin Infect Dis* 2022[67] |
| 86 | B.1.1.7  Alpha | 2021 | M/30 | DLBCL and HIV | Anti-CD20 and chemotherapy | 255 | Fever | Remdesivir | No | No | Cured | mutations in the RBD | NA | Riddell, *Clin Infect Dis*, 2022[67] |
| 87 | NA | NA | M/50 | CCL | Fludarabine, cyclophosphamide, and R | 56 | Cough and dyspnea | Remdesivir  CPT | No | No | Cured | NA | NA | Helleberg, *J Infec Dis*, 2020[68] |
| 88 | B.1 | 2020 | F/56 | FL | R bendamustine | 120 | Dyspnea and dry cough | CPT | Yes | No | Cured | 12 base-pair substitutions | NA | Reuken, *Leukaemia*, 2021[69] |

AA : amino acid, ALL : acute lymphoblastic lymphoma, AML: acute myeloid lymphoma, APLS: anti-phospholipid syndrome; ARDS : acute respiratory distress syndrome, ASCT : autologous stem cell transplant, BOOP : broncholitis obliterans with organizing pneumonia, CAPA: Covid-19 related pulmonary aspergillosis, CLL: chronic lymphocytic leukemia; COPD: chronic obstructive pulmonary disease; CPAP: continuous positive airway pressure, CPT : convalescent plasma therapy, CTC : corticosteroids, DLBCL: extranodal diffuse large B-cell lymphoma, DXM: dexamethasone; F : female, FCR : fludarabine, cyclophosphamide, rituximab, FL: follicular lymphoma; HIV: human immunodeficiency virus; HL :Hodgkin lymphoma, HSCT: hematopoietic stem cell transplantation; IL ; interleukin, IS: immunosuppressants; IVIg: intravenous immunoglobulins; F: female; M: male; MDS: myelodysplastic syndrome; M Ab: monoclonal antibodies, MDS : myelodysplastic syndrome, MIS : multisystemic inflammatory syndrome, MM: multiple myeloma; MMF : mycofenolate Mofetyl, MZL: marginal zone lymphoma; NA: not available; NHL: non Hodgkin lymphoma, NTD: N-terminal domain, ORF : open reading frame, PSCT : peripheral stem cell transplant, R: rituximab; RBD : receptor binding domain, RNA : ribonucleic acid, SCID: severe combined immune deficiency; SOT: solid organ transplantation.

**References**

1. Choi B, Choudhary MC, Regan J, et al. Persistence and Evolution of SARS-CoV-2 in an Immunocompromised Host. N Engl J Med **2020**; 383:2291–2293. Available at: http://www.nejm.org/doi/10.1056/NEJMc2031364. Accessed 31 October 2022.

2. Martinot M, Jary A, Fafi-Kremer S, et al. Emerging RNA-Dependent RNA Polymerase Mutation in a Remdesivir-Treated B-cell Immunodeficient Patient With Protracted Coronavirus Disease 2019. Clin Infect Dis **2021**; 73:e1762–e1765.

3. Avanzato VA, Matson MJ, Seifert SN, et al. Case Study: Prolonged Infectious SARS-CoV-2 Shedding from an Asymptomatic Immunocompromised Individual with Cancer. Cell **2020**; 183:1901-1912.e9.

4. Nakajima Y, Ogai A, Furukawa K, et al. Prolonged viral shedding of SARS-CoV-2 in an immunocompromised patient. Journal of Infection and Chemotherapy **2021**; 27:387–389. Available at: https://linkinghub.elsevier.com/retrieve/pii/S1341321X20304360. Accessed 16 November 2022.

5. Man Z, Jing Z, Huibo S, Bin L, Fanjun Z. Viral shedding prolongation in a kidney transplant patient with COVID‐19 pneumonia. Am J Transplant **2020**; 20:2626–2627. Available at: https://onlinelibrary.wiley.com/doi/10.1111/ajt.15996. Accessed 16 November 2022.

6. Spinicci M, Mazzoni A, Coppi M, et al. Long-term SARS-CoV-2 Asymptomatic Carriage in an Immunocompromised Host: Clinical, Immunological, and Virological Implications. J Clin Immunol **2022**;

7. Kemp S, Collier D, Datir R, et al. Neutralising antibodies in Spike mediated SARS-CoV-2 adaptation. Infectious Diseases (except HIV/AIDS), 2020. Available at: http://medrxiv.org/lookup/doi/10.1101/2020.12.05.20241927. Accessed 17 January 2021.

8. Lynch M, Macori G, Fanning S, et al. Genomic Evolution of SARS-CoV-2 Virus in Immunocompromised Patient, Ireland. Emerg Infect Dis **2021**; 27:2499–2501.

9. Baang JH, Smith C, Mirabelli C, et al. Prolonged Severe Acute Respiratory Syndrome Coronavirus 2 Replication in an Immunocompromised Patient. J Infect Dis **2021**; 223:23–27.

10. Sepulcri C, Dentone C, Mikulska M, et al. The Longest Persistence of Viable SARS-CoV-2 With Recurrence of Viremia and Relapsing Symptomatic COVID-19 in an Immunocompromised Patient—A Case Study. Open Forum Infectious Diseases **2021**; 8:ofab217. Available at: https://academic.oup.com/ofid/article/doi/10.1093/ofid/ofab217/6257145. Accessed 21 November 2022.

11. Sonnleitner ST, Prelog M, Sonnleitner S, et al. Cumulative SARS-CoV-2 mutations and corresponding changes in immunity in an immunocompromised patient indicate viral evolution within the host. Nat Commun **2022**; 13:2560. Available at: https://www.nature.com/articles/s41467-022-30163-4. Accessed 21 November 2022.

12. Tarhini H, Recoing A, Bridier-Nahmias A, et al. Long term SARS-CoV-2 infectiousness among three immunocompromised patients: from prolonged viral shedding to SARS-CoV-2 superinfection. J Infect Dis **2021**;

13. Camprubí D, Gaya A, Marcos MA, et al. Persistent replication of SARS-CoV-2 in a severely immunocompromised patient treated with several courses of remdesivir. Int J Infect Dis **2021**; 104:379–381.

14. Gandhi S, Klein J, Robertson AJ, et al. De novo emergence of a remdesivir resistance mutation during treatment of persistent SARS-CoV-2 infection in an immunocompromised patient: a case report. Nat Commun **2022**; 13:1547. Available at: https://www.nature.com/articles/s41467-022-29104-y. Accessed 31 October 2022.

15. Fourati S, Gautier G, Chovelon M, et al. Persistent SARS-CoV-2 Alpha Variant Infection in Immunosuppressed Patient, France, February 2022. Emerg Infect Dis **2022**; 28:1512–1515.

16. Adachi E, Saito M, Koga M, Tsutsumi T, Yotsuyanagi H. Favorable Outcome Following Sotrovimab Monoclonal Antibody in a Patient with Prolonged SARS-CoV-2 Omicron Infection with HIV/AIDS. Intern Med **2022**;

17. Wee LE, Tan JY, Ko KK-K, et al. Detection of viable SARS-CoV-2 in deep respiratory specimens despite negative nasopharyngeal SARS-CoV-2 RT-PCR: Occult COVID-19 as an unsuspected cause of pulmonary infiltrates in immunocompromised patients. IDCases **2022**; 30:e01611. Available at: https://linkinghub.elsevier.com/retrieve/pii/S2214250922002396. Accessed 1 November 2022.

18. Nagai H, Saito M, Adachi E, et al. Casirivimab/imdevimab for active COVID-19 pneumonia persisted for nine months in a patient with follicular lymphoma during anti-CD20 therapy. Jpn J Infect Dis **2022**;

19. Leung WF, Chorlton S, Tyson J, et al. COVID-19 in an immunocompromised host: persistent shedding of viable SARS-CoV-2 and emergence of multiple mutations: a case report. International Journal of Infectious Diseases **2022**; 114:178–182. Available at: https://linkinghub.elsevier.com/retrieve/pii/S1201971221008298. Accessed 16 November 2022.

20. Hariharan SV, Muthusamy S, Asokan SK. Persistent Viral Shedding after SARS-CoV-2 Infection in an Infant with Severe Combined Immunodeficiency. Indian J Pediatr **2022**; 89:94–94. Available at: https://link.springer.com/10.1007/s12098-021-03935-x. Accessed 16 November 2022.

21. Villaseñor-Echavarri R, Gomez-Romero L, Martin-Onraet A, et al. SARS-CoV-2 Genome Variations in Viral Shedding of an Immunocompromised Patient with Non-Hodgkin’s Lymphoma. Viruses **2023**; 15:377. Available at: https://www.mdpi.com/1999-4915/15/2/377. Accessed 9 September 2023.

22. Lindahl AL, Ahava MJ, Haukipää M, Kreivi H-R, Lipponen A, Kortela E. Successful treatment of persisting SARS-CoV-2 infection in an immunocompromised patient with repeated nirmatrelvir/ritonavir courses: a case report. Infect Dis (Lond) **2023**; 55:585–589.

23. Reddy RM, Taksande A, Lakra MS, Wanjari MB. A Rare Case of Persistent COVID-19 Infection With Aspergillosis in a 12-Year-Old Child. Cureus **2023**; 15:e33973.

24. Monrad I, Sahlertz SR, Nielsen SSF, et al. Persistent Severe Acute Respiratory Syndrome Coronavirus 2 Infection in Immunocompromised Host Displaying Treatment Induced Viral Evolution. Open Forum Infect Dis **2021**; 8:ofab295.

25. Trottier CA, Wong B, Kohli R, et al. Dual Antiviral Therapy for Persistent Coronavirus Disease 2019 and Associated Organizing Pneumonia in an Immunocompromised Host. Clinical Infectious Diseases **2023**; 76:923–925. Available at: https://academic.oup.com/cid/article/76/5/923/6771205. Accessed 10 September 2023.

26. Stanevich OV, Alekseeva EI, Sergeeva M, et al. SARS-CoV-2 escape from cytotoxic T cells during long-term COVID-19. Nat Commun **2023**; 14:149.

27. Truong TT, Ryutov A, Pandey U, et al. Increased viral variants in children and young adults with impaired humoral immunity and persistent SARS-CoV-2 infection: A consecutive case series. EBioMedicine **2021**; 67:103355.

28. Hettle D, Hutchings S, Muir P, Moran E, COVID-19 Genomics UK (COG-UK) consortium. Persistent SARS-CoV-2 infection in immunocompromised patients facilitates rapid viral evolution: Retrospective cohort study and literature review. Clin Infect Pract **2022**; 16:100210.

29. Blennow O, Vesterbacka J, Tovatt T, Nowak P. Successful Combination Treatment for Persistent Severe Acute Respiratory Syndrome Coronavirus 2 Infection. Clinical Infectious Diseases **2023**; 76:1864–1865. Available at: https://academic.oup.com/cid/article/76/10/1864/7035946. Accessed 10 September 2023.

30. Bailly B, Péré H, Veyer D, et al. Persistent Coronavirus Disease 2019 (COVID-19) in an Immunocompromised Host Treated by Severe Acute Respiratory Syndrome Coronavirus 2 (SARS-CoV-2)-Specific Monoclonal Antibodies. Clin Infect Dis **2022**; 74:1706–1707.

31. Nagasaki Y, Kadowaki M, Nakamura A, et al. A Case of a Malignant Lymphoma Patient Persistently Infected with SARS-CoV-2 for More than 6 Months. Medicina **2023**; 59:108. Available at: https://www.mdpi.com/1648-9144/59/1/108. Accessed 14 September 2023.

32. Rizzo A, Foschi A, Bracchitta F, et al. Persistent detection and sequencing of SARS-CoV-2 in the bloodstream of an immunocompromised patient. J Med Virol **2023**; 95:e28381.

33. Hirotsu Y, Kobayashi H, Kakizaki Y, et al. Multidrug-resistant mutations to antiviral and antibody therapy in an immunocompromised patient infected with SARS-CoV-2. Med **2023**; :S2666-6340(23)00257-X.

34. Zhabokritsky A, Mubareka S, Kozak RA, et al. Persistent infection with severe acute respiratory coronavirus virus 2 (SARS-CoV-2) in a patient with untreated human immunodeficiency virus (HIV). Infect Control Hosp Epidemiol **2023**; 44:350–351.

35. Brandolini M, Zannoli S, Gatti G, et al. Viral Population Heterogeneity and Fluctuating Mutational Pattern during a Persistent SARS-CoV-2 Infection in an Immunocompromised Patient. Viruses **2023**; 15:291.

36. Mhanna, Mustafa, Ismail, Alkhatatneh. Rare Case of Persistent SARS-CoV-2 for 9 Months in HIV Patient. **2023**;

37. Montejano R, Marcelo C, Falces-Romero I, et al. Efficacy of sotrovimab for persistent coronavirus disease-2019 in a severely immunocompromised person living with HIV. AIDS **2022**; 36:751–753.

38. Morel A, Imbeaud S, Scemla A, et al. Severe relapse of SARS-CoV-2 infection in a kidney transplant recipient with negative nasopharyngeal SARS-CoV-2 RT-PCR after rituximab. Am J Transplant **2022**; 22:2099–2103.

39. Ko KKK, Yingtaweesittikul H, Tan TT, et al. Emergence of SARS-CoV-2 Spike Mutations during Prolonged Infection in Immunocompromised Hosts. Microbiol Spectr **2022**; 10:e0079122.

40. Arai T, Mukai S, Kazama R, et al. Persistent viral shedding of severe acute respiratory syndrome coronavirus 2 after treatment with bendamustine and rituximab: A case report. J Infect Chemother **2022**; 28:810–813.

41. Schenker C, Hirzel C, Walti LN, et al. Convalescent plasma and remdesivir for protracted COVID-19 in a patient with chronic lymphocytic leukaemia: a case report of late relapse after rapid initial response. Br J Haematol **2022**; 196:e27–e29.

42. Cabañero-Navalon MD, Garcia-Bustos V, Ruiz-Rodriguez P, et al. Persistent SARS-CoV-2 infection with repeated clinical recurrence in a patient with common variable immunodeficiency. Clin Microbiol Infect **2022**; 28:308–310.

43. Morishita M, Suzuki M, Matsunaga A, et al. Prolonged SARS-CoV-2 infection associated with long-term corticosteroid use in a patient with impaired B-cell immunity. J Infect Chemother **2022**; 28:971–974.

44. Moutinho-Pereira S, Calisto R, Sabio F, Guerreiro L. High-titre convalescent plasma therapy for an immunocompromised patient with systemic lupus erythematosus with protracted SARS-CoV-2 infection. BMJ Case Rep **2021**; 14:e244853. Available at: https://casereports.bmj.com/lookup/doi/10.1136/bcr-2021-244853. Accessed 15 September 2023.

45. Taha Y, Wardle H, Evans AB, et al. Persistent SARS-CoV-2 infection in patients with secondary antibody deficiency: successful clearance following combination casirivimab and imdevimab (REGN-COV2) monoclonal antibody therapy. Ann Clin Microbiol Antimicrob **2021**; 20:85.

46. Nussenblatt V, Roder AE, Das S, et al. Yearlong COVID-19 Infection Reveals Within-Host Evolution of SARS-CoV-2 in a Patient With B-Cell Depletion. J Infect Dis **2022**; 225:1118–1123.

47. Drouin AC, Theberge MW, Liu SY, et al. Successful Clearance of 300 Day SARS-CoV-2 Infection in a Subject with B-Cell Depletion Associated Prolonged (B-DEAP) COVID by REGEN-COV Anti-Spike Monoclonal Antibody Cocktail. Viruses **2021**; 13:1202.

48. Keitel V, Bode JG, Feldt T, et al. Case Report: Convalescent Plasma Achieves SARS-CoV-2 Viral Clearance in a Patient With Persistently High Viral Replication Over 8 Weeks Due to Severe Combined Immunodeficiency (SCID) and Graft Failure. Front Immunol **2021**; 12:645989. Available at: https://www.frontiersin.org/articles/10.3389/fimmu.2021.645989/full. Accessed 16 September 2023.

49. Ueda Y, Asakura S, Wada S, Saito T, Yano T. Prolonged COVID-19 in an Immunocompromised Patient Treated with Obinutuzumab and Bendamustine for Follicular Lymphoma. Intern Med **2022**; 61:2523–2526. Available at: https://www.jstage.jst.go.jp/article/internalmedicine/61/16/61_9136-21/_article. Accessed 16 September 2023.

50. Shoji K, Suzuki A, Okamoto M, et al. Prolonged shedding of infectious viruses with haplotype switches of SARS-CoV-2 in an immunocompromised patient. J Infect Chemother **2022**; 28:1001–1004.

51. Bronstein Y, Adler A, Katash H, Halutz O, Herishanu Y, Levytskyi K. Evolution of spike mutations following antibody treatment in two immunocompromised patients with persistent COVID-19 infection. J Med Virol **2022**; 94:1241–1245.

52. Hanssen J, Stienstra J, Boers S, et al. Convalescent Plasma in a Patient with Protracted COVID-19 and Secondary Hypogammaglobulinemia Due to Chronic Lymphocytic Leukemia: Buying Time to Develop Immunity? Infectious Disease Reports **2021**; 13:855–864. Available at: https://www.mdpi.com/2036-7449/13/4/77. Accessed 17 September 2023.

53. Caccuri F, Messali S, Bortolotti D, et al. Competition for dominance within replicating quasispecies during prolonged SARS-CoV-2 infection in an immunocompromised host. Virus Evolution **2022**; 8:veac042. Available at: https://academic.oup.com/ve/article/doi/10.1093/ve/veac042/6590319. Accessed 17 September 2023.

54. Borges V, Isidro J, Cunha M, et al. Long-Term Evolution of SARS-CoV-2 in an Immunocompromised Patient with Non-Hodgkin Lymphoma. mSphere **2021**; 6:e0024421.

55. Peters JL, Fall A, Langerman SD, et al. Prolonged Severe Acute Respiratory Syndrome Coronavirus 2 Delta Variant Shedding in a Patient With AIDS: Case Report and Review of the Literature. Open Forum Infect Dis **2022**; 9:ofac479.

56. Scherer EM, Babiker A, Adelman MW, et al. SARS-CoV-2 Evolution and Immune Escape in Immunocompromised Patients. N Engl J Med **2022**; 386:2436–2438. Available at: http://www.nejm.org/doi/10.1056/NEJMc2202861. Accessed 10 September 2023.

57. Zimmerli A, Monti M, Fenwick C, et al. Case Report: Stepwise Anti-Inflammatory and Anti-SARS-CoV-2 Effects Following Convalescent Plasma Therapy With Full Clinical Recovery. Front Immunol **2021**; 12:613502. Available at: https://www.frontiersin.org/articles/10.3389/fimmu.2021.613502/full. Accessed 17 September 2023.

58. Pérez-Lago L, Aldámiz-Echevarría T, García-Martínez R, et al. Different Within-Host Viral Evolution Dynamics in Severely Immunosuppressed Cases with Persistent SARS-CoV-2. Biomedicines **2021**; 9:808. Available at: https://www.mdpi.com/2227-9059/9/7/808. Accessed 17 September 2023.

59. Khatamzas E, Antwerpen MH, Rehn A, et al. Accumulation of mutations in antibody and CD8 T cell epitopes in a B cell depleted lymphoma patient with chronic SARS-CoV-2 infection. Nat Commun **2022**; 13:5586.

60. Yasuda H, Mori Y, Chiba A, et al. Resolution of One-Year Persisting COVID-19 Pneumonia and Development of Immune Thrombocytopenia in a Follicular Lymphoma Patient With Preceding Rituximab Maintenance Therapy: A follow-up Report and Literature Review of Cases With Prolonged Infections. Clin Lymphoma Myeloma Leuk **2021**; 21:e810–e816.

61. Ciuffreda L, Lorenzo-Salazar JM, Alcoba-Florez J, et al. Longitudinal study of a SARS-CoV-2 infection in an immunocompromised patient with X-linked agammaglobulinemia. J Infect **2021**; 83:607–635.

62. Thornton CS, Huntley K, Berenger BM, et al. Prolonged SARS-CoV-2 infection following rituximab treatment: clinical course and response to therapeutic interventions correlated with quantitative viral cultures and cycle threshold values. Antimicrob Resist Infect Control **2022**; 11:28. Available at: https://aricjournal.biomedcentral.com/articles/10.1186/s13756-022-01067-1. Accessed 17 September 2023.

63. Berktas BM, Koyuncu A. Case Report: Unremitting COVID-19 Pneumonia, Viral Shedding, and Failure to Develop Anti-SARS-CoV-2 Antibodies for More Than 6 Months in Patient with Mantle Cell Lymphoma Treated with Rituximab. Am J Trop Med Hyg **2022**; 106:1104–1107.

64. Purpura LJ, Chang M, Annavajhala MK, et al. Prolonged severe acute respiratory syndrome coronavirus 2 persistence, attenuated immunologic response, and viral evolution in a solid organ transplant patient. American Journal of Transplantation **2022**; 22:649–653. Available at: https://linkinghub.elsevier.com/retrieve/pii/S1600613522081084. Accessed 17 September 2023.

65. Gibson EG, Pender M, Angerbauer M, et al. Prolonged SARS-CoV-2 Illness in a Patient Receiving Ocrelizumab for Multiple Sclerosis. Open Forum Infectious Diseases **2021**; 8:ofab176. Available at: https://academic.oup.com/ofid/article/doi/10.1093/ofid/ofab176/6214941. Accessed 17 September 2023.

66. Hensley MK, Bain WG, Jacobs J, et al. Intractable Coronavirus Disease 2019 (COVID-19) and Prolonged Severe Acute Respiratory Syndrome Coronavirus 2 (SARS-CoV-2) Replication in a Chimeric Antigen Receptor-Modified T-Cell Therapy Recipient: A Case Study. Clinical Infectious Diseases **2021**; 73:e815–e821. Available at: https://academic.oup.com/cid/article/73/3/e815/6122591. Accessed 17 September 2023.

67. Riddell AC, Kele B, Harris K, et al. Generation of Novel Severe Acute Respiratory Syndrome Coronavirus 2 Variants on the B.1.1.7 Lineage in 3 Patients With Advanced Human Immunodeficiency Virus-1 Disease. Clin Infect Dis **2022**; 75:2016–2018.

68. Helleberg M, Niemann CU, Moestrup KS, et al. Persistent COVID-19 in an Immunocompromised Patient Temporarily Responsive to Two Courses of Remdesivir Therapy. J Infect Dis **2020**; 222:1103–1107.

69. Reuken PA, Stallmach A, Pletz MW, et al. Severe clinical relapse in an immunocompromised host with persistent SARS-CoV-2 infection. Leukemia **2021**; 35:920–923. Available at: https://www.nature.com/articles/s41375-021-01175-8. Accessed 17 September 2023.

70. Lee CY, Shah MK, Hoyos D, et al. Prolonged SARS-CoV-2 Infection in Patients with Lymphoid Malignancies. Cancer Discov **2022**; 12:62–73.
